# Healthcare Workers’ Strategies for Doffing Personal Protective Equipment

**DOI:** 10.1093/cid/ciz613

**Published:** 2019-09-13

**Authors:** Jure Baloh, Heather Schacht Reisinger, Kimberly Dukes, Jaqueline Pereira da Silva, Hugh P Salehi, Melissa Ward, Emily E Chasco, Priyadarshini R Pennathur, Loreen Herwaldt

**Affiliations:** 1 Department of Internal Medicine, University of Iowa, Iowa City; 2 Department of Health Policy and Management, University of Arkansas for Medical Sciences, Little Rock; 3 Center for Access and Delivery Research and Evaluation, Iowa City VA Health Care System, Iowa City; 4 Institute for Clinical and Translational Science, University of Iowa, Iowa City; 5 Department of Industrial and Systems Engineering, University of Iowa, Iowa City; 6 Department of Engineering Management, Systems and Technology, University of Dayton, Ohio; 7 Department of Epidemiology, University of Iowa, Iowa City

**Keywords:** healthcare workers, personal protective equipment, doffing, strategies

## Abstract

**Background:**

Personal protective equipment (PPE) helps protect healthcare workers (HCWs) from pathogens and prevents cross-contamination. PPE effectiveness is often undermined by inappropriate doffing methods. Our knowledge of how HCWs approach doffing PPE in practice is limited. In this qualitative study, we examine HCWs’ perspectives about doffing PPE.

**Methods:**

Thirty participants at a Midwestern academic hospital were recruited and assigned to 1 of 3 doffing simulation scenarios: 3 mask designs (n = 10), 2 gown designs (n = 10), or 2 glove designs (n = 10). Participants were instructed to doff PPE as they would in routine practice. Their performances were video-recorded and reviewed with participants. Semistructured interviews about their doffing approaches were conducted and audio-recorded, then transcribed and thematically analyzed.

**Results:**

Three overarching themes were identified in interviews: doffing strategies, cognitive processes, and barriers and facilitators. Doffing strategies included doffing safely (minimizing self-contamination) and doffing expediently (eg, ripping PPE off). Cognitive processes during doffing largely pertained to tracking contaminated PPE surfaces, examining PPE design cues (eg, straps), or improvising based on prior experience from training or similar PPE designs. Doffing barriers and facilitators typically related to PPE design, such as PPE fit (or lack of it) and fastener type. Some participants also described personal barriers (eg, glasses, long hair); however, some PPE designs helped mitigate these barriers.

**Conclusions:**

Efforts to improve HCWs’ doffing performance need to address HCWs’ preferences for both safety and expediency when using PPE, which has implications for PPE design, training approaches, and hospital policies and procedures.

## Teaser

Healthcare workers seek both safety and expediency when doffing personal protective equipment (PPE). While doffing, they track contaminated surfaces, follow design cues, and rely on experience to improvise. They encounter numerous doffing barriers and facilitators, chiefly pertaining to PPE design.

Effective use of personal protective equipment (PPE) by healthcare workers (HCWs) is an important component of infection prevention in healthcare settings. PPE (eg, gowns, gloves, masks) protects HCWs from contamination with infectious agents and helps prevent cross-contamination to other patients. However, PPE effectiveness is influenced by how HCWs wear and doff (remove) PPE, which was highlighted prominently by the recent outbreak of Ebola virus disease (EVD) [1].

Despite wearing PPE for their safety, HCWs routinely self-contaminate while doffing PPE, with self-contamination rates as high as 46%–90% across PPE types (eg, gowns, gloves) and scenarios [2–4]. For example, Kwon et al [5] found that among HCWs asked to doff either contact precautions or EVD PPE, 26% and 44% contaminated themselves, respectively. Casanova et al [6] found contamination on 53% of inner gloves and 13% of scrubs worn by HCWs trained specifically in EVD PPE. In an observational study, Kang et al [3] estimated that 66% of HCWs potentially contaminated themselves when doffing PPE after caring for patients in isolation precautions.

Contamination commonly results from critical doffing errors [2, 7–10], even when HCWs believe they are proficient in doffing [11]. When HCWs doff complex PPE for a high-risk scenario such as EVD, they often make errors during key “vulnerable processes,” including when reaching for equipment during the doffing process and when removing respirators and hoods [8–10]. During more routine contact precautions, HCWs often self-contaminate when removing gloves [2, 12] and gowns [7, 13]. Several factors may contribute to self-contamination, including poorly fitting or “universally sized” PPE [14–16], difficulty distinguishing between dirty (outside) and clean (inside) surfaces while doffing [14], and forceful or rushed movements [14, 17]. HCWs may use incorrect doffing sequences or methods that do not work well with the PPE design [17]. Furthermore, doffing protocols are often not standardized [15], and HCWs may receive suboptimal and inconsistent PPE training [18], leading to further confusion regarding appropriate doffing approaches.

Our understanding of doffing practices and factors that contribute to self-contamination is growing but is largely based on examination of PPE doffing protocols for high-consequence infectious agents, such as Ebola virus. Furthermore, we have limited knowledge about HCWs’ perceptions and thoughts about doffing PPE. In this study, we focused on HCWs’ perceptions of doffing PPE used in routine care, and we examined how HCWs approach doffing, what they think about and pay attention to while doffing, and what factors facilitate or inhibit their ability to doff.

## METHODS

### Study Design and Context

This qualitative study was part of a larger simulation study to investigate doffing practices with methods that include task analysis, contamination count and location analysis, eye tracking, basic anthropometry measurements, and participant think-aloud interviews. We briefly describe the larger study to provide context. All findings reported here are based on qualitative participant interviews; findings that pertain to other data collected will be reported in future articles. The study was reviewed and approved by the University of Iowa Institutional Review Board.

### Sample and Data Collection

We recruited 30 HCWs and students (medical and nursing students doing clinical rotations who use PPE) at a large teaching hospital in the Midwestern United States. We excluded faculty and staff who did not have clinical patient care duties and students who were not doing clinical rotations. After obtaining written consent, we conducted anthropometry measurements and asked participants to fill out a demographics questionnaire.

Participants were assigned to 1 of 3 doffing simulation scenarios. The first 10 participants used standard gloves and 3 mask designs: procedure mask with ear loops, surgical mask with ties, and pouch-style mask with headbands. The next 10 participants used standard gloves and 2 gown designs: over-the-head isolation gown with break-away neck closure and thumb loops and isolation gown with tape-tab neck closure and elastic cuffs. The last 10 participants used 2 glove designs: standard nonsterile nitrile exam gloves and Doffy gloves (nonsterile exam gloves with a Doffy flap, a doffing aid positioned in the wrist area [19]). We examined the participants with a black light to assess baseline contamination, asked them to don the PPE items, and sprayed them with Glo Germ fluorescent marker. We then instructed participants to doff PPE as they would in practice. Because participants were not familiar with Doffy gloves, we showed them the Doffy flap and explained that it was to help them doff the gloves. After participants doffed the PPE, we reexamined them with a black light to assess self-contamination and asked them to thoroughly wash their hands and faces. The study was conducted in a simulation room where the whole sequence was video-recorded from 4 angles.

We reviewed the video recordings with the participants and conducted short (typically about 10 minutes) semistructured interviews using the retrospective think-aloud method [20, 21]. In these interviews, we asked participants open-ended questions to explain their doffing approach and to share what they were thinking about or paying attention to at each step in the process. We paused or rewound the video, asked follow-up probing questions, and reviewed the donning process as necessary. The interviews were audio-recorded (after obtaining verbal consent) and transcribed verbatim.

### Qualitative Analyses

We analyzed the transcripts using both a priori (deductive) and emergent (inductive) codes [22]. The a priori codes were overarching themes that reflected our research questions, and the emergent codes were subthemes identified within each overarching theme. One investigator (J. B.) started developing and refining the codebook based on a sample of transcripts and met periodically with 2 medical anthropologists on the team (H. S. R., K. D.) to discuss and refine the themes. After the codebook was finalized, J. B. coded the remaining transcripts. To further enhance reliability, a second coder (J. P. S.) randomly selected and coded 2 transcripts from each simulation scenario (6 total, or 20% of all transcripts). The 2 coders met to compare the coded content and found that the codebook did not need to be revised. They discussed coding differences until they reached agreement. After coding the overarching themes, J. B. examined the coded content within each theme to identify and enumerate the most prominent response patterns, which he compared and grouped together into subthemes using comparative analyses [23]. Finally, to identify any systematic differences, we determined whether occurrence of subthemes varied across PPE types and designs and by HCW type.

## RESULTS

Characteristics of the participants recruited are summarized in Table 1. We identified 3 overarching interview themes: doffing strategies, cognitive processes, and barriers and facilitators. Doffing strategies focused on how participants generally approached doffing PPE. Cognitive processes focused on what participants thought about or paid attention to while doffing PPE, chiefly reflecting how they carried out the strategies or how they overcame unanticipated issues and doffing barriers. Barriers and facilitators addressed factors that helped or hindered doffing (eg, PPE design, personal factors). Table 2 includes exemplar quotes to illustrate each theme.

**Table 1.** Characteristics of Study Participants

Characteristic	By Simulation Scenario			Total (N = 30)
	Mask + Gloves (n = 10)	Gown + Gloves (n = 10)	Gloves only (n = 10)	
Age (mean, range)	43, 20–62	29, 21–52	36, 25–66	36, 20–66
Sex (% female)	70	90	80	80
Healthcare Worker Type (n)				
Nurse	3	6	6	15
Physician	3	1	2	6
Student	1	3	1	5
Other	3^a^	0	1^b^	4
Years of experience (n)				
Less than 1	0	2	1	3
1–5	2	5	3	10
5–10	3	1	3	7
>10	5	2	3	10
Prior training (%)				
Personal protective equipment donning and doffing	90	80	90	87
Hand hygiene	100	100	100	100

^a^Nursing assistant, clinical pharmacist, and respiratory therapist.

^b^Physician assistant.

**Table 2.** Exemplar Quotes for Interview Themes

Theme	Exemplar quotes
Doffing strategies	• I don’t want to contaminate. When taking off the first glove, you want to go on the outside of the glove and pull it off without touching. And the other glove, you want to go inside and pull it off, so you don’t get contaminated. (nurse)
	• [I’m] trying to have the least amount of contact with the exterior of either mask…just hold it away and get it off without touching. (respiratory therapist)
	• I just start pulling it, the gown, because I always take the gown off first. But, that leaves for splashing, because it splashed my face…. So maybe next time I need to actually untie it, instead of just ripping it. (nurse)
	• I think I was a little bit more careful than when you’re just exiting the room, you kind of just rip it off. (student)
	• I had trouble undoing the knot, I was just going to tear it, but I got it out finally. I would normally tear it off….I get frustrated and, you know, I’m kind of busy, so I don’t want to sit there futzing with it. (physician)
Cognitive processes	• I was making sure I was touching the inside part [of the gown]. I guess I could have made a mistake and touched the outside part, but I didn’t feel like I did. I was paying attention not to do that. (nurse)
	• I was wondering what the tabs [flaps on Doffy gloves] were for and I thought, “Oh, perhaps a tab is to pull them.” (physician)
	• In class we were trained to just rip it [the gown] off. But with this one I felt, since it was tied in the front, that I needed to untie it first. And since the other one was tied in the back, I figured it would be easier [to] tear it off. (student)
	• I saw the mask, I realized that there was only one strap and it was in the center, so it wasn’t like an N-95 where you can actually cup it in your hand and hold it on your face and then pull the strings back. So I improvised. (physician)
	• I was confused because of the whole different gown. I wasn’t sure how I was supposed to get it off in the correct way. (nurse)
	• I think the first [set of gloves] I didn’t think through, I just sort of wanted to do it by muscle memory. (nurse)
Barriers and facilitators	• I thought that maybe I’m contaminating my neck, because it was more difficult for me to remove the knot behind my head. (physician)
	• With the headband I wasn’t sure if I could pull it to break it. (clinical pharmacist)
	• The belt was a little bit thicker, so it was harder to just pull it off. (student)
	• This is the knot that I can’t see, but I tied it the same way as the other one, with the single pull to release. (nurse)
	• I usually don’t use them [gown straps] because then--, well, I’m trying to stay clean, and pulling them off is more of a process, to try and stay clean… I feel like it’s, like, trying to untie it would be more apt to contaminate the backside. (nurse)
	• Try to figure out a way to get it to not splash. [I: Mm-hmm.] But it’s so hard to when you have to rip it. So the force of it can make something fly off the gown. (nurse)
	• It’s the most difficult part for me…. You try not to contaminate, but then your glasses are there and you don’ know what to do. (physician)
	• If I get my arms in there [inside the gown] it gives me a little bit more mobility….If it’s on there, you can almost grip it from the inside, cause they’re so big. They’re big on everybody, they’re not going to fit me. (student)
	• Then I start at the bottom, and then normally I try and grab it and see if I can get the gown to do this, but the gowns were too tight, I couldn’t get it to [rip]. (nurse)
	• The tab [Doffy flap] was nice, but I didn’t know how I would use it for the second one [glove]. (nurse)
	• I still think that the most user friendly was the yellow one [procedure mask], with the little strings [ear loops], and because I’m familiar with it. (nurse)

In the following sections we describe each theme and its subthemes and we highlight differences across PPE types and designs and by HCW type (as applicable). We also include the number of participants who discussed each subtheme (in parentheses); however, the numbers are provided for illustrative purposes only and should not be used for statistical inference. Furthermore, comparison across HCW types should be interpreted with caution because the sample size was small, different groups of HCWs were unequally represented in the 3 doffing scenarios (which could bias group comparisons), and since the subthemes emerged later in our analyses, we did not solicit responses that pertained to specific subthemes during the interviews (ie, absence of evidence is not evidence of absence).

### Doffing Strategies

We identified 2 subthemes of doffing strategies: doffing safely and doffing expediently. Most participants (n = 29) described trying to doff safely and minimize the likelihood of self-contamination, primarily by avoiding contact with the surfaces they perceived as contaminated (eg, front of the gown, outside surface of the gloves) and using gestures to better control PPE (eg, not ripping PPE off or ripping in a careful, controlled manner). About half of the participants (n = 17) also described doffing expediently, which involved approaches to doffing PPE quickly and with minimal burden (eg, ripping PPE off) or using shortcuts and work-arounds (often initiated at donning; eg, not fastening gown straps). Participants who described both strategies did not specify which strategy was their priority, but some noted that doffing expediently could undermine safety.

We found that response patterns for doffing strategies varied across PPE types and designs. In the mask simulation scenario, the tension between safety and expediency tended to be most pronounced for the surgical mask with ties (which can be ripped off). For example, several participants tried to untie the mask because they thought it would be safer to remove that way, though some admitted that in practice they would probably rip the mask off. However, most participants described using both strategies to doff gowns and typically did not see much conflict or tension between them. While ripping gowns was not perceived as inherently unsafe, some participants described difficulties ripping gowns off in a controlled manner or described other barriers (see Cognitive Processes and Barriers and Facilitators). For gloves, most participants described only doffing safely; they mentioned expediency (or lack thereof) only in reference to alternative glove doffing methods (eg, the “beak method”), which they rarely use in routine practice. We also found that doffing expediently was less commonly reported by physicians (2/6) than other HCWs (15/24).

### Cognitive Processes

Participants described several things they thought about or paid attention to when doffing (and also donning) PPE. Most commonly (n = 20) they described tracking PPE surfaces they thought were most likely contaminated and trying not to touch them. About half of the participants (n = 14) also described looking for PPE design cues (eg, the type of fastener used), often starting this process while donning the PPE. These cues helped suggest the optimal doffing method, particularly when the HCW was not familiar with the PPE item. In unforeseen circumstances (eg, doffing unfamiliar PPE designs, encountering doffing barriers), participants also tended to improvise approaches that made sense to them, typically relying on training or experience with similar PPE designs (n = 7).

Participants’ responses regarding cognitive processes did not vary notably across different PPE types and designs. Tracking contaminated surfaces and looking for design cues were more common with gowns than with masks and gloves, and improvising was more common with masks and gowns than with gloves. Four participants described relying on muscle memory and not thinking much at all when doffing gloves. Furthermore, we found that tracking PPE surfaces was more commonly reported by students (5/5) than other HCWs (15/25) and that looking for PPE design cues was also more commonly reported by students (4/5) than other HCWs (10/25).

### Barriers and Facilitators

Doffing barriers and facilitators typically pertained to PPE design. Participants identified the fasteners (eg, bands, straps) on masks and gowns as particularly problematic. For example, a common barrier was the (perceived) need to untie certain mask and gown designs (n = 14). Knots were often behind participants’ heads or backs, making them hard to find or hard to reach (particularly for HCWs with mobility issues). Sometimes knots were also hard to untie. Furthermore, when untied, the loose fasteners or the PPE itself could be hard to control and could pose a contamination risk. Several participants described work-arounds that prevented or minimized the problem of untying PPE (n = 5), such as tying just 1 knot, not tying the fasteners while donning, or simply ripping the PPE off rather than untying the fasteners. However, ripping off PPE was not easy with certain designs (n = 7), particularly the masks with bands and the gowns with thicker belts. Some participants also found the gown with the tape-tab neck closure harder to rip off in a predictable and controlled way. While gloves were not associated with the barriers described above, several participants (n = 6) described difficulty safely doffing the glove on the second hand (because the other hand was exposed). PPE fit was also a barrier to doffing all types of PPE (n = 10), typically when PPE fit too tightly. However, while 2 participants found tighter-fitting gowns harder to doff, 2 participants found looser-fitting gowns harder to doff.

Participants also identified personal barriers (n = 10), such as wearing personal items (glasses, watches), having long hair, and having mobility issues. However, some PPE designs helped mitigate these barriers. For example, procedure masks with ear loops were the easiest to doff for participants who wore glasses. Some participants also admitted that they struggled to recall the correct doffing approach, but they generally described remembering or “figuring out” how to doff correctly. Finally, 3 participants explicitly commented on the benefit of using familiar PPE designs because it allowed them to rely on their doffing habits.

## Discussion

In this study, we examined how HCWs perceive and think about doffing PPE in routine care settings. Our findings indicate that HCWs seek a balance of safety and expediency when doffing PPE. While doffing, they track and avoid contact with PPE surfaces they think are likely contaminated. When doffing unfamiliar PPE items or in other unforeseen circumstances, HCWs tend to improvise and rely on PPE design cues, prior training, and experience with similar PPE. We identified several factors that facilitated or impeded doffing, most of which were related to PPE design, such as type and location of fastener and fit.

HCWs are often aware that they violate doffing protocols [17, 24] and are cognizant of the trade-offs between safety and expediency. HCWs in clinical practice are busy and use PPE frequently, which likely influences them to doff more expediently than they would otherwise. They may be more likely to doff expediently in routine care, which they perceive to be low risk, particularly if they see PPE as self-protection rather than a measure to prevent cross-contamination [24, 25]. These findings suggest that efforts to improve PPE use and doffing should address both safety and expediency through new and improved PPE designs, doffing methods, training approaches, and organizational policies and procedures.

We found some differences between groups of HCWs. Physicians were less likely to report doffing expediently, which could either mean they are less likely to acknowledge during an in-person interview that they doff expediently or that doffing expediently is less salient to them. The difference in salience could be due to differences in clinical practice workflows. For example, Harrod et al found that during routine care, physicians (and physical therapists) don and doff PPE much less frequently than nurses [26]. This suggests that different HCWs groups have unique PPE needs and challenges and that each group may require a training program tailored to their clinical practice. We also found that students were more likely to report tracking potentially contaminated PPE surfaces and looking for PPE design cues. While other HCW types may have simply omitted this information from their responses (eg, it may be self-evident to them that one would do those things while doffing), it may also suggest that more experienced HCWs have internalized cognitive processes associated with specific tasks and, thus, may perform tasks such as PPE doffing without consciously processing relevant information, relying rather on muscle memory and habits.

Our findings indicate that PPE should be redesigned in ways that facilitate expedient and safe doffing methods. Design cues could help HCWs envision and execute appropriate doffing methods; for example, color-coding PPE surfaces to differentiate “dirty” outside surfaces from “clean” inside surfaces could help HCWs track where these surfaces are at each step of the doffing process [16, 27]. Fasteners (eg, straps, belts) that do not require untying and are easy to undo (or rip) quickly would facilitate speed and ease of doffing [16, 27]. PPE also must fit people of different sizes and physical abilities and should be either adjustable or available in several sizes [14, 27]. In addition, new doffing methods and additional training may be required to optimize HCWs’ doffing of redesigned PPE. The new PPE designs and doffing methods also must be rigorously tested, not only for efficacy in “ideal” and controlled settings but also for effectiveness in practice. If HCWs use work-arounds to avoid doffing PPE with cumbersome but safe methods, it may be prudent to design PPE that leads to fewer work-arounds and allows for better compliance with doffing methods in practice, even if such designs and methods are shown to be somewhat less safe in controlled settings. In addition to efficacy and effectiveness studies, well-designed ethnographic and human factors approaches can help us better understand the variety of factors that influence HCWs’ ability to doff in routine clinical practice, where and why critical errors occur, and how to improve usability of PPE and doffing methods.

New training approaches may help improve HCWs’ doffing performance [2, 18]. Our findings indicate that HCWs draw on prior training experiences when encountering new PPE designs or facing doffing barriers, suggesting that PPE training should cover the range of PPE types and designs HCWs may encounter in clinical practice and the most appropriate doffing methods for each. HCWs should also be given just-in-time training if their standard PPE is replaced with a new style during shortages or vendor changes. Furthermore, HCWs could benefit from training that would help them assess how to doff unfamiliar PPE or improvise solutions when they encounter doffing barriers or PPE failures (eg, if the gown shreds into multiple pieces). New training approaches must be tested for effectiveness [18]. While computerized training may be prevalent [18], HCWs may prefer hands-on practical training [24], which would also allow for feedback on self-contamination with fluorescent markers [2] and would allow HCWs to develop muscle memory. HCWs at different stages (eg, in-school vs on-the-job training) may also need different training approaches. Thus, training protocols should accommodate HCWs’ level of experience and the work they perform.

Finally, hospitals and other healthcare organizations can play an important role in implementing needed changes and sustaining high performance over time by providing necessary support [3, 16, 28]. Hospitals should consider assembling interdisciplinary teams to periodically review and revise their PPE protocols [16]. PPE protocols must be clear and unambiguous in order to provide guidance for key processes, from PPE selection to its use in practice. After carefully examining their PPE needs, hospitals could eliminate unnecessary variation in PPE designs to minimize the likelihood that HCWs would encounter unfamiliar PPE, and they could procure PPE that is safe and expedient to use and doff. Furthermore, hospitals could tailor their PPE training approaches for the PPE available at their institution in order to increase the likelihood that HCWs use appropriate doffing methods. HCWs may require refresher training, particularly if new PPE designs are introduced into practice or they start working in new settings that use different PPE designs. To maintain or improve performance over time, hospitals should monitor compliance with PPE use and doffing protocols and investigate reasons for poor performance.

Our study had several limitations. First, all participants were recruited at 1 teaching hospital and its affiliated schools. Common influences (eg, institutional policies and procedures, training, available PPE) may have limited the range of perspectives we identified in the study. However, participants’ work experience (in years) varied substantially. Thus, participants likely were initially trained at different times, in different institutions, and with different PPE designs, which should mitigate this limitation. Second, the simulation context of our study (eg, presence of observers, simulation room, recording, fluorescent marker, other prompts) may have influenced participants to pay closer attention to doffing and particularly to avoiding self-contamination, leading them to adopt different doffing approaches than they use in practice, or bias their interview responses. Furthermore, in a simulation study, we could not account for the range of tasks and circumstances that HCWs encounter in day-to-day practice but may influence doffing performance (eg, carrying objects while doffing [17]). Thus, we may have not explored the full range of factors that influence HCWs’ PPE doffing practice.

## Conclusions

This study contributes to the literature on PPE doffing performance and self-contamination by focusing on HCWs’ perspectives. HCWs try to balance safety and expediency when doffing PPE, which triggers various cognitive processes during doffing. Different PPE designs can facilitate or inhibit HCWs’ doffing practices. These findings have implications for designing the next generation of PPE and new doffing methods, developing and implementing PPE training protocols, and revising policies and procedures in hospitals and other healthcare organizations.

## Notes

***Acknowledgments.*** The authors thank Sandra Cobb for contributing to this research. Halyard provided gowns, gloves, and masks. IP Gloves GmbH proved the Doffy gloves. Neither company had any input into the study design, data collection, data interpretation, or manuscript preparation.

***Financial support.*** This work was supported by the Centers for Disease Control and Prevention (CK000448-01). J. B. acknowledges support from the National Institute on Drug Abuse (T32DA022981).

***Supplement sponsorship.*** This article appears as part of the supplement “Personal Protective Equipment for Preventing Contact Transmission of Pathogens: Innovations from CDC’s Prevention Epicenters Program,” sponsored by the CDC’s Prevention Epicenters Program.

***Potential conflicts of interest.*** All authors report no potential conflicts. All authors have submitted the ICMJE Form for Disclosure of Potential Conflicts of Interest. Conflicts that the editors consider relevant to the content of the manuscript have been disclosed.
